# A case of *Wohlfahrtiimonas chitiniclastica* bacteremia in continental United States

**DOI:** 10.1099/jmmcr.0.005134

**Published:** 2017-12-21

**Authors:** Jesus A. Chavez, Andrew J. Alexander, Joan M. Balada-Llasat, Preeti Pancholi

**Affiliations:** ^1^​ Department of Pathology, The Ohio State University Wexner Medical Center, Columbus, Ohio, USA; ^2^​ Department of Internal Medicine, Division of Infectious Diseases, The Ohio State University Wexner Medical Center, Columbus, Ohio, USA

**Keywords:** *Wohlfahrtiimonas chitiniclastica*, infection, bacteremia, MALDI-TOF MS, ulcer, osteomyelitis, antibiotics, susceptibility

## Abstract

**Introduction:**

*Wohlfahrtiimonas chitiniclastica* has been associated with open wound infections, cellulitis, osteomyelitis, and bacteremia.

**Case presentation:**

We report the case of a 41 year old woman with history of congenital myelomeningocele, paraplegia and extensive decubitus ulcers that developed *W. chitiniclastica* bacteremia secondary to an infected ulcer.

**Conclusion:**

*W. chitiniclastica* is an emerging human pathogen that can be readily identified by MALDI-TOF or sequencing.

## Introduction

The first description of *Wohlfahrtiimonas chitiniclastica* was made in 2008 by Tóth *et al*. and the name of the bacteria is related to the first described vector, a species of obligate parasitic flies known as *Wohlfahrtia magnifica* [1]. It is known to be transmitted by other fly species, including *Chrysomya megacephala*, *Lucilia sericata* and *Musca domestica*, when larvae are deposited in open wounds [2]. *W. chitiniclastica* is a strictly aerobic, non-motile, non-spore forming, Gram-negative rod that grows best between 28 and 37 ˚C. It is catalase- and oxidase-positive, but urease-, indole- and H_2_S-negative [1]. A strong chitinase activity has been described as an important characteristic [1, 3]. It has been reported to be misidentified by the VITEK 2 system, but successfully confirmed by matrix-assisted laser desorption ionization–time of flight mass spectrometry (MALDI-TOF MS) and 16S rRNA gene sequencing [2, 4]. A total of twelve cases of human infection have been described in the literature worldwide [2] and, even though it has been mostly associated with parasitic flies, the bacteria have also been isolated in chicken meat [5] and arsenic-affected soils in Bangladesh [6]. The majority of the reports have been from areas with relatively warm climates, but there is a recent case report from an area of northern temperate climate [3, 7]. Here we present the first, to our knowledge, well documented case of bacteremia and the second case of human infection in the continental USA.

## Case report

A 41-year-old female presented to the emergency department with a one-week history of abdominal pain, worsening back pain and constipation. Past medical history included congenital lumbar myelomeningocele causing paraplegia status post spinal fixation, extensive sacral decubitus ulcers, obesity, severe lower extremity lymphedema, Arnold Chiari malformation type II with remote ventriculoperitoneal shunt placement and neurogenic bladder with chronic indwelling Foley catheter. She complained of vomiting and fatigue but denied fevers and chills. Her Foley catheter was changed two days prior to admission. Upon physical examination, she was found to have a foul smelling stage IV right ischial decubitus ulcer, bilateral leg lymphedema with multiple skin excoriations and poor dentition. The patient was unable to recall how long she had the wounds. The wounds had worsened over time due to inadequate wound care and the inability to shower at home due to body habitus and paraplegia. Additional exposure history included living with a dog and cigarette smoking. She denied intravenous (IV) drug use.

**Fig. 1.**
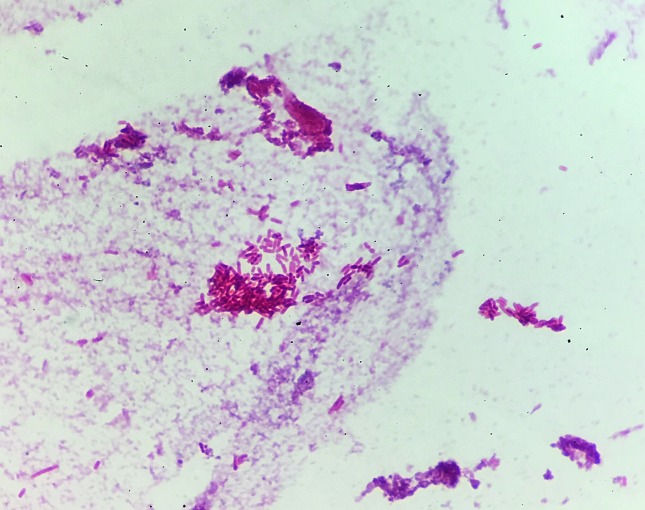
Gram-negative rod from positive blood culture on microscopic examination (1000×).

**Fig. 2.**
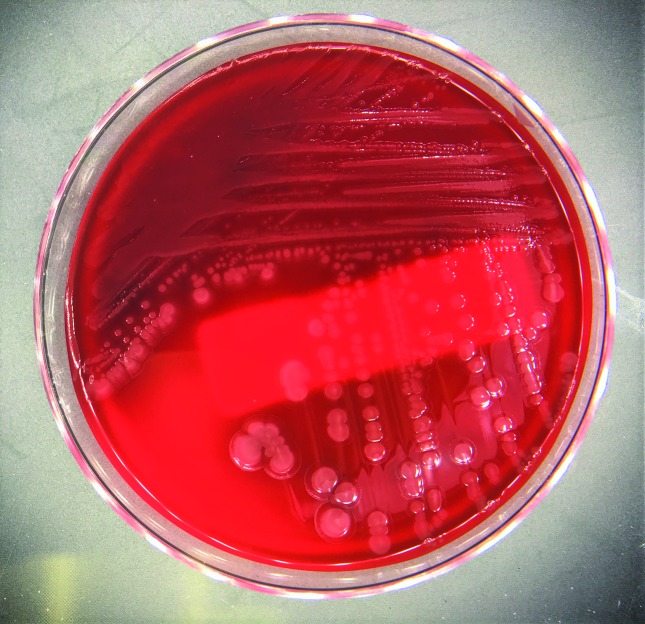
Colonies growing on sheep blood agar revealing a characteristic mucoid morphology.

Laboratory data was consistent with an elevated white blood cell count (WBC) of 42.60 K µl^−1^, hyperkalemia, hyponatremia and pyuria with acute kidney injury. Computed tomography of the abdomen and pelvis was concerning for osteomyelitis of the right ischium with adjacent abscess. The patient was diagnosed with sepsis, blood cultures were obtained and she was treated with empiric IV vancomycin and cefepime. An unsuccessful attempt to place an intraosseous catheter was performed. Blood culture became positive after 17 h of incubation and a Gram stain of the blood culture revealed Gram-negative bacilli (Fig. 1). The sample was inoculated to sheep blood, chocolate and MacConkey media (Becton, Dickinson and Company) and cultured aerobically at 37 ˚C for 24 h, growing *W. chitiniclastica* (Fig. 2). *Proteus mirabilis* was also isolated from the culture after 48 h. The organisms were identified accurately by MALDI-TOF MS (RUO version 3.1, Bruker Daltonics) with a score of >2.0. Extraction was not required. A culture sent from the ischial wound grew *W. chitiniclastica*, *Myroides injenensis* and *Enterococcus faecalis* aerobically, and *Bacteroides ovatus*/*Bacteroides xylanisolvens* anaerobically. Susceptibilities were determined using the MicroScan WalkAway (Beckman Coulter). The interpretation of antibiotic susceptibility was based on the minimal inhibitory concentration breakpoints (µg ml^−1^) for other non-Enterobacteriaceae described in the M100 Performance Standards for Antimicrobial Susceptibility Testing, CLSI, 27^th^ edition January 2017. Appropriate quality controls (*Escherichia coli* ATCC 35218, *Klebsiella pneumoniae* ATCC 700603 and *Pseudomonas aeruginosa* ATCC 27853) were utilized for the interpretation of susceptibility results. The *W. chitiniclastica* isolate was susceptible to all antibiotics tested (Table 1).

**Table 1.** Antimicrobial susceptibility testing of *W. chitiniclastica*

Antibiotic	MIC (µg ml^−1^)	Susceptible=S
Amikacin	≤16	S
Cefepime	≤4	S
Ceftazidime	≤1	S
Ciprofloxacin	≤1	S
Gentamicin	≤4	S
Levofloxacin	≤2	S
Piperacillin–tazobactam	≤16	S
Tobramycin	≤4	S
Trimethoprim–sulfamethoxazole	≤2/38	S

The patient remained afebrile throughout her admission and WBC improved with antibiotics. On hospital day 6 she was taken to the operating room for wound debridement, but no additional cultures were obtained. Given her improvement on hospital day 15 she was discharged to a skilled nursing facility on IV vancomycin and cefepime via a peripherally inserted central catheter, and oral metronidazole for a planned 6 week course.

Five days after discharge she developed altered mental status and became hypotensive. She was admitted to the ICU and required vasopressor support. She was treated with IV vancomycin and meropenem. A stool PCR was positive for *Clostridium difficile* so IV metronidazole and oral vancomycin were added. She developed worsening metabolic acidosis, requiring continuous renal replacement therapy, and acute encephalopathy, necessitating intubation for airway protection. Despite aggressive treatment she continued to decline and on hospital day three, her family decided to transition her to comfort care and she expired shortly thereafter. Blood cultures from the second admission were initially negative, but a line culture was positive for *Candida albicans* shortly after she expired.

## Discussion

We present the fifth case of *W. chitiniclastica* bacteremia worldwide [2, 3, 7–9], the first bacteremia case and second of human infection in the continental USA. The first reported case involved an open leg wound [4]. Other cases in the USA have been described, including a zoonotic case of bacterial septicemia in Michigan in 2014 [10], and two elderly patients infected in Hawaii [11], one of whom was bacteremic. To the best of our knowledge, a total of 12 cases of human disease [2–4, 7–9, 12] and three zoonotic cases [10, 13, 14] have been reported in the literature. Misidentification as *Acinetobacter iwoffii*, *Comamonas testosteroni* or *Rhizobium radiobacter* has been reported in previous cases using the Vitek 2 system [2, 4, 7]. When our isolate was tested with the MicroScan WalkAway, the system was unable to identify the organism since low probability scores for *Moxarella/Psychrobacter* spp., *Vibrio* spp., *Pasteurella/Actinobacillus* spp. and *Pseudomonas* spp. were generated (data not shown). Neither of the systems (Vitek and Microscan) includes these organisms in their database. MALDI-TOF MS and 16S rRNA sequencing have proven to be good identification methods for *W. chitiniclastica* [2, 7, 8, 11, 15], in fact, the *W. chitiniclastica* strain SH04 genome is available [16].

The geographic distribution of *W. chitiniclastica* is variable, but most cases have been described in Europe [2–4, 7–9, 11, 12], however, epidemiological studies are required to better understand its geographic distribution and host-associated risk factors. To date, all reported cases of human infections are related to poor hygienic conditions and/or chronic open skin wounds with multiple comorbidities. Several cases have been associated with maggots. Our patient did not have any evidence of larvae or maggots but did have chronic wounds with very poor wound care and hygiene. Continuous exposure of the open wound to the environment might have facilitated transmission through exposure to *Musca domestica* or other fly species. Nevertheless, this cannot be proven with certainty since the patient denied any other risk factors, including recent travel history, and maggots were not identified by physical exam or autopsy. The vast majority of infections are polymicrobial, including our case, in which *Proteus mirabilis* bacteremia was also identified, albeit 24 h after *W. chitiniclastica* was isolated, thus we consider *W. chitiniclastica* to be the primary pathogen. The organism has been shown to be susceptible to the majority of the antimicrobials available, with β-lactams being the most commonly used treatment. Intrinsic fosfomycin resistance has been reported [2, 5].

In conclusion, *W. chitiniclastica* is an emerging human pathogen, sometimes associated with flies and maggots, which often causes infection of chronic wounds especially in the setting of poor hygienic conditions. Both MALDI-TOF MS and 16S rRNA sequencing are able to identify this organism reliably.

## References

[1] Tóth EM, Schumann P, Borsodi AK, Kéki Z, Kovács AL (2008). *Wohlfahrtiimonas chitiniclastica* gen. nov., sp. nov., a new gammaproteobacterium isolated from *Wohlfahrtia magnifica* (Diptera: Sarcophagidae). Int J Syst Evol Microbiol.

[2] Schröttner P, Rudolph WW, Damme U, Lotz C, Jacobs E (2017). *Wohlfahrtiimonas chitiniclastica*: current insights into an emerging human pathogen. Epidemiol Infect.

[3] Rebaudet S, Genot S, Renvoise A, Fournier PE, Stein A (2009). *Wohlfahrtiimonas chitiniclastica* bacteremia in homeless woman. Emerg Infect Dis.

[4] de Dios A, Jacob S, Tayal A, Fisher MA, Dingle TC (2015). First report of *Wohlfahrtiimonas chitiniclastica* isolation from a patient with cellulitis in the United States. J Clin Microbiol.

[5] Matos J, Queiroga AP, de Oliveira Pedroza Bindi dos Reis CC, Ribeiro RL, Teixeira LM (2016). First report of the emerging zoonotic agent *Wohlfahrtiimonas chitiniclastica* isolated from a retail frozen chicken in Rio de Janeiro, Brazil. Antonie Van Leeuwenhoek.

[6] Sanyal SK, Mou TJ, Chakrabarty RP, Hoque S, Hossain MA (2016). Diversity of arsenite oxidase gene and arsenotrophic bacteria in arsenic affected Bangladesh soils. AMB Express.

[7] Kõljalg S, Telling K, Huik K, Murruste M, Saarevet V (2015). First report of *Wohlfahrtiimonas chitiniclastica* from soft tissue and bone infection at an unusually high northern latitude. Folia Microbiol.

[8] Almuzara MN, Palombarani S, Tuduri A, Figueroa S, Gianecini A (2011). First case of fulminant sepsis due to *Wohlfahrtiimonas chitiniclastica*. J Clin Microbiol.

[9] Campisi L, Mahobia N, Clayton JJ (2015). *Wohlfahrtiimonas chitiniclastica* bacteremia associated with Myiasis, United Kingdom. Emerg Infect Dis.

[10] Thaiwong T, Kettler NM, Lim A, Dirkse H, Kiupel M (2014). First report of emerging zoonotic pathogen *Wohlfahrtiimonas chitiniclastica* in the United States. J Clin Microbiol.

[11] Nogi M, Bankowski MJ, Pien FD (2016). *Wohlfahrtiimonas chitiniclastica* Infections in 2 elderly patients, Hawaii. Emerg Infect Dis.

[12] Suryalatha K, John J, Thomas S (2015). *Wohlfahrtiimonas chitiniclastica*-associated osteomyelitis: a rare case report. Future Microbiol.

[13] Josue DD, Eva S, Isabel VA, Lucas D, Marisa A (2015). Endocarditis associated with *Wohlfahrtiimonas chitiniclastica* in a short-beaked common dolphin (*Delphinus delphis*). J Wildl Dis.

[14] Qi J, Gao Y, Wang GS, Li LB, Li LL (2016). Identification of *Wohlfahrtiimonas chitiniclastica* isolated from an infected cow with hoof fetlow, China. Infect Genet Evol.

[15] Schröttner P, Gunzer F, Schüppel J, Rudolph WW (2016). Identification of rare bacterial pathogens by 16S rRNA gene sequencing and MALDI-TOF MS. J Vis Exp.

[16] Cao XM, Chen T, Xu LZ, Yao LS, Qi J (2013). Complete genome sequence of *Wohlfahrtiimonas chitiniclastica* strain SH04, isolated from *Chrysomya megacephala* collected from Pudong International Airport in China. Genome Announc.

